# Magnetically Repulsive Cushion Triboelectric Nanogenerator for Rotating Machinery Structural Health Monitoring

**DOI:** 10.3390/s26113587

**Published:** 2026-06-04

**Authors:** Haojie Peng, Yufen Wu, Yanling Li, Yingjie He, Changke Wang, Xin Na, Qiang Tan, Wei Qiu, Xiaohong Yang

**Affiliations:** 1 College of Physics and Optoelectronic Engineering, Chongqing Normal University, Chongqing 401331, China; 2023051102031@cqnu.edu.cn (H.P.); 2023051102034@stu.cqnu.edu.cn (C.W.); 2 College of Optoelectronic Engineering, Chongqing University, Chongqing 400044, China; 3 Chongqing Municipal Water Supply Co., Ltd., Chongqing 400060, China; cqnaxin@163.com (X.N.); tanqiang727@163.com (Q.T.); cqzlsqw@163.com (W.Q.)

**Keywords:** triboelectric nanogenerator, magnetically repulsive cushion, microstructured strip electrode array, CNN-GRU, rotating machinery health monitoring

## Abstract

**Highlights:**

**What are the main findings?**

- A magnetically repulsive cushion triboelectric nanogenerator enables self-powered vibration sensing under weak excitation.
- The combined magnetic cushioning and microstructured strip electrodes improve contact–separation stability, output performance, and signal reliability.

**What are the implications of the main findings?**

- The proposed MRCT provides a low-power sensing strategy for rotor vibration monitoring without an external power supply.
- A CNN-GRU model enables laboratory-scale recognition of predefined rotor imbalance states, achieving over 98% accuracy under the present dataset.
- The MRCT also shows distinguishable time- and frequency-domain responses under a shaft-misalignment-related abnormal vibration condition.

**Abstract:**

Rotor imbalance and abnormal vibration are classical operating conditions in rotating machinery and can often be identified by conventional vibration analysis. However, the development of low-power, self-powered, and distributed sensing nodes remains important for long-term condition monitoring, particularly in scenarios where external power supply, wiring, and maintenance are constrained. Existing vibration sensors, including piezoelectric and capacitive types, are constrained by power consumption and degraded performance under low-frequency and weak excitation. To address this issue, a magnetically repulsive cushion triboelectric nanogenerator (MRCT) is proposed to enable self-powered vibration sensing. The magnetic-repulsion cushion allows the upper friction layer to undergo stable contact–separation motion under a non-contact restoring force, while the microstructured strip electrode array (MSEA) enhances the triboelectric output and signal stability. A hybrid convolutional neural network–gated recurrent unit (CNN-GRU) deep-learning model is employed to extract time-domain and frequency-domain features from the collected signals, enabling real-time identification of rotor vibration amplitude, frequency, and imbalance weight. Experimental results show that the MRCT provides stable output, a high signal-to-noise ratio, and an identification accuracy above 98% for predefined rotor imbalance-weight states under laboratory conditions. In addition, a shaft-misalignment-related abnormal vibration condition was examined on the motor platform. The corresponding time-domain and frequency-domain analyses show that the MRCT voltage signal exhibits distinguishable signal variations under normal and misalignment-related conditions, including spectral changes around the 2× rotational frequency. A laboratory-scale AIoT-oriented demonstration further verifies the feasibility of integrating MRCT signal acquisition, CNN-GRU inference, wireless transmission, and GUI-based visualization. It should be noted that the present work mainly focuses on imbalance-state recognition, while the misalignment-related experiment provides an additional sensor-response verification. Broader validation involving mechanical looseness, bearing defects, variable-speed operation, cross-machine testing, and long-term industrial conditions remains necessary.

## 1. Introduction

As industrial intelligence continues to advance worldwide, rotating machinery plays an increasingly important role in manufacturing, transportation, wind energy, and emerging power-generation systems. The operational state of such machinery is closely tied to production efficiency and equipment safety [1]. Yet high rotational speed and complicated service conditions often lead to rotor imbalance, vibration-induced fatigue, and component wear, which create substantial challenges for maintenance and reliability control [2]. These issues call for vibration-sensing technologies that combine high sensitivity, low-power consumption, and real-time monitoring capability, thereby enabling predictive maintenance and intelligent health management for rotating machinery [3,4].

Rotor imbalance usually leads to obvious vibration, and the information embedded in these vibration signals can be used to characterize the operating condition of machinery. Precise acquisition and analysis of such signals make it possible to evaluate rotor status and identify faults in real time. This also indicates that research on mechanical vibration signals provides the theoretical basis for improving vibration sensor performance. To realize intelligent recognition of these vibrations, a range of energy harvesting and sensing mechanisms has been investigated, mainly including piezoelectric approaches [5,6,7] and triboelectric approaches [8,9,10]. In practical use, conventional accelerometers and piezoelectric sensors are still constrained by high power demand, complicated installation, and limited environmental adaptability [11]. Triboelectric nanogenerators (TENGs), in contrast, can directly convert mechanical vibration into electrical signals and feature simple structures, easy miniaturization, stable output, and high sensitivity. These merits make TENGs attractive for vibration monitoring in rotating machinery [12].

Recent progress has confirmed the applicability of TENGs in road vibration monitoring [13,14,15], seismocardiographic and phonocardiographic sensing [16,17,18], machine tactile sensing [19,20,21], and pipeline damage detection [22]. Most traditional TENG-based vibration sensors depend on spring-assisted or cantilever configurations, where power enhancement is achieved by increasing the motion amplitude [23,24]. For example, Wang et al. reported a dual-spring TENG for highly linear vibration-frequency measurement within 0–200 Hz [25]. Tang et al. proposed a cantilever-based two-degree-of-freedom TENG, in which the system resonance frequency was optimized by adjusting beam stiffness [26]. Despite these advances, such structures still show limited bandwidth in complex vibration environments, strong sensitivity to excitation direction, and difficulty maintaining stable output under low-frequency nonlinear vibration [27].

To address these issues, researchers have introduced non-contact magnetic designs to optimize TENG performance [28]. Yang et al. developed a magnetofluid-tuned TENG, in which the natural frequency was regulated by a magnetic field to improve output performance [29]. Ding et al. reported a magnetic-field-assisted TENG that significantly enhanced output capability and multidirectional response by modulating the motion trajectory through magnetic force [30]. Building on these efforts, this work introduces a non-contact cushioning layer based on magnetic repulsion and further incorporates a microstructured strip electrode array (MSEA) [31,32]. Compared with conventional spring-based designs, the proposed structure provides wider frequency adaptability, more stable output, and stronger capability for extracting mechanical-state information in complex vibration environments [33].

As industrial equipment becomes more digitalized, vibration monitoring is increasingly shifting toward data-driven intelligent methods. Deep learning is well-suited to extracting informative features from complex vibration signals and, compared with conventional threshold-based approaches, often delivers better accuracy and stronger robustness. When TENG sensors are integrated with deep learning and IoT platforms, vibration monitoring can be performed more efficiently and intelligently, with reduced demands on manual maintenance and long-term tracking [34,35]. Zhao et al., for instance, designed a flexible coaxial fiber-shaped TENG vibration sensor and applied deep learning to classify its electrical output, achieving highly accurate identification of nine operating conditions [36]. Chen et al. likewise developed a TENG sensor for rail vibration energy harvesting and realized high-accuracy classification of vibration states through deep learning [37]. Since TENG was first proposed by Zhong Lin Wang in 2012, growing attention has been directed toward its combination with artificial intelligence. Although reports on rotor imbalance detection are still limited, TENG-based sensors are likely to play a more important role in intelligent industrial monitoring [38,39]. On this basis, the present study combines TENG sensing with deep learning for accurate, real-time recognition of vibration states and aims to provide a reliable strategy for intelligent industrial monitoring.

Despite the growing use of TENGs in vibration monitoring, existing strategies still face several limitations. First, conventional spring-assisted and cantilever-based structures usually have restricted adaptability and narrow bandwidth under low-frequency excitation. Second, deep-learning methods often perform well only when the differences among signal patterns are sufficiently obvious, which limits their real-time diagnostic performance. Third, direct utilization of TENG output in industrial inspection remains technically challenging. To overcome these issues, this work develops an MRCT. By introducing a magnetic-repulsion cushion, the upper friction layer is driven into stable periodic motion through a non-contact restoring force. Meanwhile, the MSEA is incorporated to enlarge the effective triboelectric contact area and improve charge-transfer efficiency, leading to enhanced output voltage and vibration sensitivity. A CNN-GRU model is then employed to extract time- and frequency-domain features from the acquired signals for real-time identification of rotor vibration amplitude, frequency, and imbalance weight. After the sensor node is integrated into an industrial monitoring system, the machine state can be visualized in real time through a graphical interface, enabling remote monitoring and early warning of rotating-machinery health conditions. Experimental results indicate that the MRCT delivers stable electrical signals under different excitation conditions and can accurately capture vibration features associated with rotor imbalance. The device also shows a high signal-to-noise ratio and good frequency-tracking behavior. Coupled with the deep-learning model, the proposed system achieves more than 98% accuracy in rotor-state recognition. These results demonstrate the feasibility of using the MRCT as a self-powered vibration-sensing node for laboratory-scale rotor imbalance-state recognition and AIoT-oriented monitoring. The proposed strategy provides a complementary sensing route for low-power and distributed rotating-machinery condition monitoring, while further industrial validation is still required for practical deployment. In addition to rotor imbalance, a shaft-misalignment-related abnormal vibration condition is considered to further examine the fault-response capability of the MRCT. In practical rotating machinery, shaft misalignment can alter the dynamic response of the rotor system and is often accompanied by harmonic variations, particularly around the 2× rotational frequency. Therefore, the misalignment-related vibration signals are analyzed in both the time and frequency domains to evaluate whether the MRCT can capture fault-related spectral variations beyond the vibration-intensity changes caused by rotor imbalance.

Rotor imbalance is a classical vibration-related condition in rotating machinery and can often be detected by conventional acceleration measurement and frequency-domain analysis. Therefore, this study does not aim to argue that the imbalance itself is difficult to diagnose. Instead, the problem addressed here is the sensing architecture for distributed, low-power, and difficult-to-access monitoring scenarios. As summarized in Table S1 in the Supporting Information, capacitive, piezoelectric, piezoresistive, and triboelectric accelerometer sensors have different advantages in sensitivity, flexibility, and self-powered operation. Commercial and MEMS accelerometers provide mature vibration measurement, but they generally require an external power supply, readout circuits, and wiring. Triboelectric sensors can generate electrical signals directly from mechanical vibration, making them suitable for self-powered vibration monitoring where wiring and long-term maintenance are limiting factors.

## 2. Materials and Methods

### 2.1. Design and Working Principle of the MRCT

**Figure 1:**
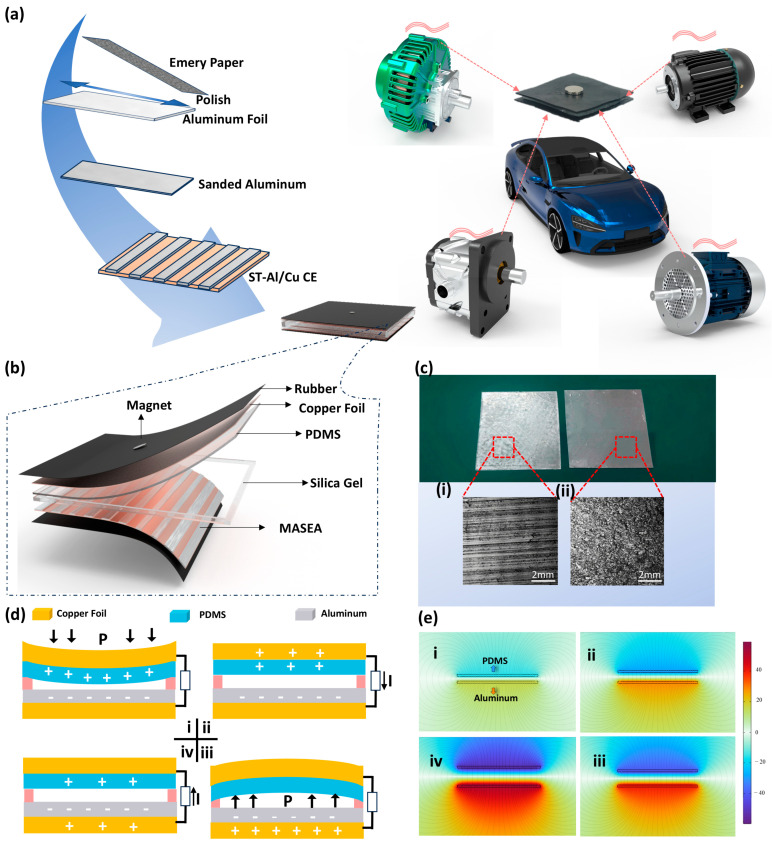
Application scenarios, structure, and working principle of the MRCT. (**a**) Schematic diagram of the MRCT application scenario and the manufacturing process of MSEA. (**b**) Structure of the MRCT. (**c**) Comparison of microstructured aluminum foil and regular aluminum foil: (**i**) microstructured aluminum foil (Sanded Aluminum) after treatment; (**ii**) untreated aluminum foil. (**d**) Working mechanism of the MRCT; the black arrows indicate the direction of external pressure/excitation, and the arrows between panels indicate the contact–separation cycle: (**i**) initial contact state under external pressure; (**ii**) separation state after pressure release; (**iii**) reverse deformation state under upward excitation; (**iv**) restored separation state during the contact–separation cycle. (**e**) COMSOL Multiphysics (v6.3) simulation of the potential variation during electrode contact–separation in the MRCT: (**i**) initial contact state; (**ii**) early separation state; (**iii**) increased separation state; (**iv**) maximum separation state.

Figure 1a shows the fabrication route of the MSEA and its potential applications in robots, vehicles, and industrial equipment. Commercial aluminum foil was first cut into the required dimensions and then roughened with sandpaper to produce sanded aluminum with a microstructured surface. The foil was then cut into strips and arranged on a copper-foil substrate with a preset spacing to form the MSEA. Figure 1b presents the configuration of the triboelectric sensor built on the MSEA. The sensor is composed of magnets, PDMS, the MSEA, silicon, and other functional materials. The key structural parameters of the MRCT were measured and are summarized in Table S2 in the Supporting Information. The magnets had dimensions of [5 mm × 0.5 mm/diameter × thickness], and the initial distance between the opposing magnets was [20 mm]. The PDMS layer had a thickness of [0.05 mm], an effective contact area of [400 mm^2^], and was prepared with a base-to-curing-agent ratio of [1:1]. These structural parameters directly affect the vibration response and electrical output of the MRCT. In this structure, the magnets play a central role. By generating magnetic repulsion, they create a cushioning layer that introduces a non-contact restoring force between the friction layer. This force replaces conventional mechanical elastic elements and also regulates the friction layer contact and separation during vibration. In this way, excessive contact can be avoided, impact can be reduced, and stable contact–separation motion can be maintained under external excitation.

Figure 1c(i) corresponds to a conventional planar aluminum electrode. Compared with this planar electrode, the aluminum electrode fabricated by the present facile method, as shown in Figure 1c(ii), significantly increases the effective contact area, enhances local deformation, and creates more contact–separation interfaces. These structural advantages markedly improve triboelectric output and vibration sensitivity, providing a basis for the subsequent sensor design. Figure 1e illustrates the operating principle of the MRCT. Under external vibration, magnetic repulsion drives the friction layer into periodic contact and separation. During this process, triboelectrification occurs between the PDMS friction layer and the surface of the microstructured aluminum electrode, resulting in equal and opposite charges on the two surfaces. When the friction layer separates, the interfacial potential difference induces electron flow in the external circuit, generating a current signal and converting mechanical vibration into electrical output. At the same time, the non-contact restoring force supplied by the magnets stabilizes the contact–separation process, improves vibration sensitivity, and enhances signal stability, thereby enabling continuous monitoring of motor vibration. Figure 1e shows the potential distribution obtained from the COMSOL simulation. The simulated results clearly reveal the potential evolution of the sensor under different operating states and offer valuable support for further design optimization.

### 2.2. Theoretical Model of Vibration Response and Triboelectric Output

To clarify the factors that dominate the output behavior of the MRCT and to evaluate its vibration-response characteristics, theoretical analysis was performed from two aspects: vibration dynamics and triboelectric energy conversion. Considering the structural features of the device, the system under external vibration can be simplified as a forced mass-spring-damper model. The magnetic repulsive force generated by the magnets acts as a non-contact restoring force and, together with the structural elasticity, determines the effective stiffness of the system.

The dynamic response of the system is governed by the classical vibration equation shown in Equation (1):

(1) $$m\ddot{x}+c\dot{x}+k_{eff}x=F\left(t\right)$$

where m denotes the equivalent mass, c the damping coefficient, x the vibration displacement, and F(t) the external excitation. The effective stiffness $k_{eff}$ is determined by both the structural stiffness k and the magnetic-repulsion stiffness $k_{m}$, as given in Equation (2):

(2) $$k_{eff}=k+k_{m}$$

According to vibration theory, the natural frequency of the system is expressed as Equation (3):

(3) $$f_{n}=\frac{1}{2\pi }\sqrt{\frac{k_{eff}}{m}}$$

By introducing magnets and tuning their repulsive interaction, the effective stiffness of the system can be adjusted, which in turn changes the vibration response of the device. When the external excitation frequency approaches the natural frequency, the vibration displacement increases markedly, thereby strengthening the contact–separation motion between the friction layer.

The electrical output of the TENG is produced by the combined effects of contact electrification and electrostatic induction. When the PDMS friction layer contacts the microstructured aluminum electrode, opposite charges with equal magnitude are generated at the interface because of the difference in electron affinity between the two materials. As the friction layer separates under vibrational excitation, the interfacial distance x increases and a potential difference is established. According to the theoretical model of TENG [40], the open-circuit voltage is given by Equation (4):

(4) $$V=\frac{\sigma x}{\varepsilon_{0}}$$

where $\varepsilon_{0}$ is the vacuum permittivity. As the vibration displacement changes continuously, the interfacial potential difference also varies, driving electron transfer through the external circuit and generating a current signal. The short-circuit current can be expressed as Equation (5):

(5) $$I=\frac{dQ}{dt}$$

where *Q* represents the amount of transferred charge flowing through the external circuit, with the unit of coulomb (C), and *t* is time, with the unit of second (s). Thus, the short-circuit current *I* is defined as the rate of transferred charge with respect to time, with the unit of ampere (A). Periodic contact and separation induced by external vibration produce the electrical output of the device. Dynamic-response analysis under different excitation conditions yielded the displacement-response curves and spectral characteristics of the sensor (Figures S1 and S2 in the Supporting Information). The results indicate that the output frequency of the device closely matches the input vibration frequency. With increasing vibration frequency, the device response gradually approaches the input sinusoidal waveform, demonstrating good frequency-tracking performance and linear-response capability. This behavior suggests that the proposed structure can respond accurately to external vibration variations and is well-suited for practical vibration-sensing applications.

The mathematical model established in this section is used as a physical interpretation model rather than as a direct numerical prediction model for the CNN-GRU classifier. Specifically, Equations (1)–(3) describe the vibration response of the MRCT under external excitation and explain how the magnetic repulsive force and effective stiffness affect the contact-separation motion. Equations (4) and (5) further describe how vibration-induced displacement changes the interfacial potential difference and charge-transfer process, thereby determining the amplitude and periodicity of the output electrical signal. Therefore, this model provides the physical basis for the time-domain and frequency-domain signal analyses in Section 3.4 and supports the use of MRCT output signals as diagnostic features for imbalance-state recognition.

### 2.3. Experimental Setup and Signal Acquisition

**Figure 2:**
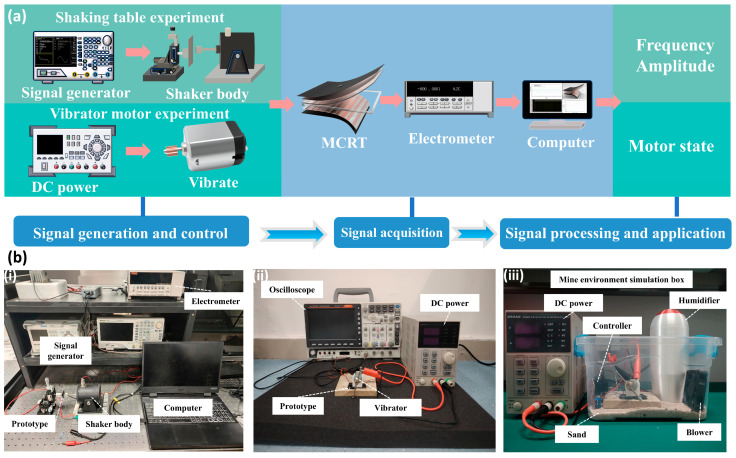
Experimental process and platform setup. (**a**) Experimental process. (**b**) Setup of vibration screen and vibration motor experiments: (**i**) setup of the vibration table performance experiment platform; (**ii**) state detection of the rotating motor under an imbalance weight; (**iii**) state detection of the rotating motor in a simulated real-world application scenario.

As illustrated in Figure 2, a set of experiments was carried out using an electrodynamic shaker (SA-JZ020, Wuxi Shi’ao Technology Co., Ltd., Wuxi, China) and a vibration motor (purchased from Taobao Marketplace, Hangzhou, China) to assess the performance of the MRCT and to examine its potential for detecting imbalance weights in electric-vehicle drive systems. The shaker tests were mainly used to evaluate the electrical output characteristics of the MRCT and to demonstrate its real-time monitoring performance under different amplitudes and frequencies. In the vibration-motor tests under imbalance-weight conditions, the operating condition of the motor was identified by monitoring its vibration response, which further confirmed the practical applicability of the MRCT. As shown in Figure 2a, the overall experimental process consisted of three stages: signal generation and control, signal acquisition, and signal processing and application.

In the signal generation and control stage, the electrodynamic shaker and the vibration motor served as two different vibration sources. In the shaker-based tests, the vibration was controlled by the input signal supplied by a signal generator, while in the motor-based tests, the vibration amplitude was adjusted using a DC power supply (KA3005D, Dongguan KORAD Technology Co., Ltd., Dongguan, China). In the shaker experiments, the excitation amplitude refers to the peak-to-peak voltage of the sinusoidal electrical signal supplied by the function generator to the shaker and is denoted as mVpp. To keep the MRCT firmly in place during measurement, it was attached to the motor under test with transparent adhesive tape. A dedicated motor clamp with bolt-mounting holes was also designed to improve structural stability and suppress external noise. For signal acquisition, only the motor clamp was used to secure the motor so as to maintain signal integrity. In the signal processing and application stage, the raw MRCT signals were denoised and filtered to obtain clearer waveforms and better signal quality. The processed data were then used for electrical analysis and deep-learning model training to extract the effective information contained in the signals.

In the shaker experiments (Figure 2b(i)), the vibration platform was driven by a sinusoidal input signal with tunable frequency and amplitude generated by the signal generator. This setup was used to evaluate the electrical output characteristics of the MRCT over a range of excitation frequencies and amplitudes, allowing its performance under different test conditions to be systematically analyzed. In the vibration-motor experiments (Figure 2b(ii)), a real-time monitoring platform based on the MRCT was constructed. The vibration motor was secured to a 300 mm × 300 mm × 25 mm wooden board using a motor clamp. To reduce vibration transmission to the ground, the motor and its base were placed on a sponge pad. Vibration was controlled by adjusting the operating condition of the DC motor, while the MRCT was mounted on the base plate. An oscilloscope (GDS-1104B, Good Will Instrument Co., Ltd., New Taipei City, Taiwan) was used to monitor the signal waveforms, and an electrometer (Model 6517A, Keithley Instruments, Inc., Solon, OH, USA) was employed to acquire the electrical signals generated by the MRCT and transfer them to a computer for subsequent data processing and deep-learning analysis.

A shaft-misalignment-related abnormal vibration condition was also examined on the same motor platform. Since the present setup used a compact DC motor rather than a coupled motor–shaft–load system, the misalignment-related condition was simulated by applying a controlled angular mounting deviation to the motor. No imbalance weight was attached during this measurement, so that the vibration response induced by the angular deviation could be evaluated separately from the rotor mass imbalance. The MRCT mounting position and signal-acquisition settings were kept the same as those used in the imbalance experiments. The acquired voltage signals were then analyzed in the time domain and frequency domain, with particular attention to the spectral region around the 2× rotational frequency.

To further assess the effect of temperature, humidity, and environmental noise on MRCT performance in a realistic setting, a wind-sand environment simulation chamber was designed (Figure 2b(iii)). The chamber reproduced the temperature and humidity conditions associated with a wind-sand environment, making it possible to evaluate the electrical behavior of the MRCT under more complex conditions. Additional environmental simulation tests were also performed to verify the reliability of the device under different environmental conditions.

The motor platform was also used to examine a shaft-misalignment-related abnormal vibration condition. Because a compact DC motor was used instead of a coupled motor–shaft–load system, this condition was simulated by applying a controlled angular mounting deviation to the motor. No imbalance weight was attached during this measurement, so that the vibration response induced by the angular deviation could be evaluated separately from the rotor mass imbalance. The MRCT mounting position and signal-acquisition settings were kept the same as those used in the imbalance experiments.

### 2.4. Data Processing and CNN-GRU Model

Guided by the physical model in Section 2.2, combined time-domain and frequency-domain analyses were performed to characterize the MRCT output signals. The results show that the output signals can effectively reflect several physical characteristics of the monitored object, particularly vibration amplitude and frequency. On this basis, a deep-learning framework combining a convolutional neural network (CNN) and gated recurrent unit (GRU) was developed for real-time vibration-state monitoring and analysis. The system is able to identify vibration amplitude and frequency in real time and to further determine the corresponding imbalance weight. Because the MRCT can capture vibration signals generated under different imbalance-weight conditions, it provides effective responses to different excitation states and improves system reliability in practical applications.

To present the monitoring results more directly, a graphical user interface (GUI) was developed to display both the raw signals and the recognition results. After training, the model can classify the measured signals, determine the level of imbalance weight, and infer its impact on device operation. The identified information is then transmitted to the user through serial communication. During deep-learning training, vibration signals corresponding to six imbalance-weight conditions were collected. Each raw acquisition was performed at a sampling rate of 100 Hz for 60 s, resulting in 6000 raw data points. The raw signals were then segmented using a sliding-window method, and each segmented window sample contained 1000 data points. Therefore, the 6000 data points refer to one raw acquisition record, whereas the 1000 data points refer to one segmented input sample for the CNN-GRU model.

To clarify the data partitioning strategy and reduce data leakage caused by highly overlapping sliding windows, each segmented sample retained the ID of its original acquisition batch. After slicing and labeling, the dataset was split using a stratified group-wise strategy based on the original acquisition batches, rather than by random window-level splitting. Specifically, samples generated from the same raw acquisition record were assigned to only one subset and were not shared among the training, validation, and test sets. The dataset was divided into training, validation, and test sets at a ratio of 70:15:15, while maintaining the class distribution of different imbalance-weight and vibration-frequency conditions in each subset. Therefore, the data were segregated at the experimental acquisition-batch level. However, rotational-speed or vibration-frequency conditions were not used as independent leave-one-speed-out test batches; instead, their distributions were kept balanced among the three subsets. All data were acquired using the same MRCT–DC motor experimental platform; therefore, equipment-batch segregation was not performed in this study. In addition, time-period segregation was considered at the raw acquisition-record level because windows from the same continuous acquisition record were not split across different subsets. Long-term cross-day or cross-period generalization was not specifically evaluated and will be investigated in future work.

**Figure 3:**
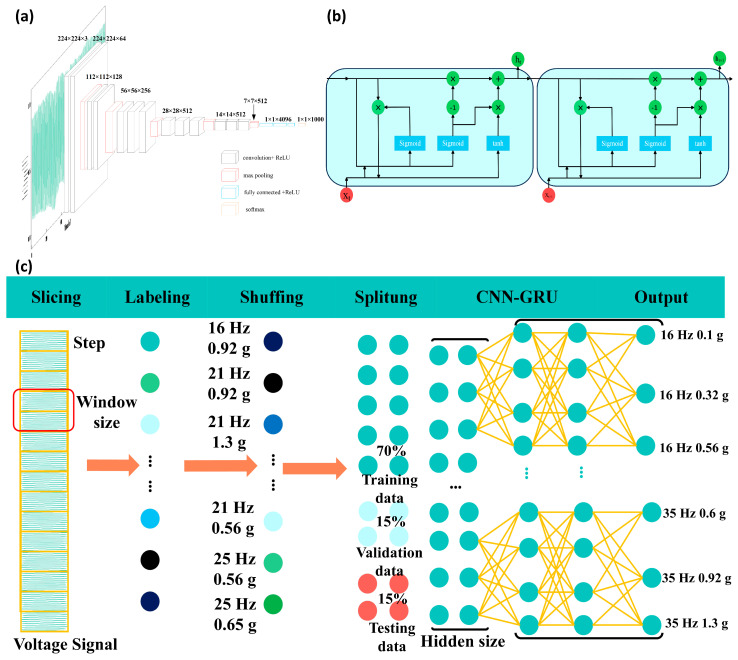
Principle of the real-time vibration-monitoring platform based on CNN-GRU. (**a**) Detailed structure of the CNN model. (**b**) Detailed structure of the GRU model. (**c**) Workflow for processing the collected MRCT voltage signals for CNN-GRU-based vibration-state recognition. The segmented samples were labeled, shuffled, and divided into training, validation, and test sets using a stratified group-wise splitting strategy based on the original acquisition batches.

Figure 3a presents the CNN architecture, while Figure 3b shows the structure of the GRU network. In this work, a deep-learning model combining CNN and GRU modules was established. The CNN is first used to automatically extract local features from the time-domain vibration signals through convolution kernels. By sliding across the signal sequence, it captures variations in amplitude, periodicity, and nonlinear waveform characteristics under different imbalance-weight conditions. This strategy avoids the uncertainty associated with hand-crafted features in traditional feature engineering and improves the model’s ability to represent complex vibration patterns. On this basis, a GRU module was introduced to process the temporal sequence features. Compared with conventional recurrent neural networks (RNNs), the GRU can capture long-range temporal dependencies more efficiently through its gating mechanism, while using fewer parameters and converging faster during training. These characteristics make it suitable for continuous vibration-signal analysis.

Through the combination of CNN and GRU, the model can extract both local signal features and temporal evolution patterns, which allows effective discrimination among vibration responses generated by different imbalance weights. Deep-learning methods of this type have already shown good performance in rotating-machinery fault diagnosis. CNNs are able to learn representative features directly from raw vibration signals, whereas recurrent structures such as GRU can further characterize the temporal dynamics of the signal sequence, thereby improving recognition accuracy. In this study, the proposed MRCT vibration-sensing system effectively distinguishes vibration features associated with different imbalance weights and provides reliable support for intelligent diagnosis and real-time monitoring of motor operating states. The experimental results further show that the method maintains high recognition accuracy under complex vibration conditions, demonstrating its potential as a practical solution for TENG-based vibration monitoring in rotating-machinery health monitoring.

The data-processing procedure is shown in Figure 3c. A sliding-window method was first used to segment the raw MRCT voltage signals, where each window contained 1000 data points, and the stride was set to 2 points. Each segmented sample was labeled according to its corresponding imbalance-weight and vibration-frequency condition and retained the ID of its original acquisition batch. After labeling, the samples were shuffled and divided into training, validation, and test sets using a stratified group-wise splitting strategy. This strategy ensured that samples derived from the same raw acquisition record were not distributed across different subsets, thereby reducing data leakage caused by the high overlap between adjacent windows. The segmented vibration signals were then input into the CNN-GRU model for feature extraction and vibration-state recognition. The segmented vibration signals were next input into a CNN for feature extraction. Using convolution kernels to scan the time-series signals locally, the CNN extracted variations in amplitude, periodicity, and nonlinear waveform characteristics under different imbalance conditions. Based on the features learned by the CNN, a bidirectional gated recurrent unit (BiGRU) layer was further introduced for temporal modeling. Owing to its gating mechanism, the GRU can effectively capture long-range dependencies in sequential data. Compared with conventional RNNs, it also requires fewer parameters and converges more rapidly, which makes it suitable for learning the dynamic characteristics of vibration signals. To alleviate overfitting, a dropout layer with a rate of 0.2 was inserted before the fully connected layer. The output layer adopted a fully connected structure for vibration-state classification and prediction of the imbalance-weight parameter. Additional regularization was also applied to the output layer to further improve generalization. The network was trained for 30 epochs, with 30 samples processed in each epoch.

## 3. Results

### 3.1. Electrical Output Performance of the MRCT

The MRCT is mainly designed for monitoring vibrations induced by rotor imbalance in motors. This type of vibration is generally associated with rotational speed and is typically manifested as a periodic excitation signal that is close to a sinusoidal waveform under steady operating conditions. For this reason, a function generator was used in the experimental setup to output sinusoidal signals that simulate the vibration input produced during motor operation. Considering the driving capability and stable working range of the vibration system, a maximum input excitation voltage of 300 mVpp was selected for testing. This choice ensured the stability and repeatability of the applied vibration excitation and helped verify the applicability of the MRCT to imbalance-vibration detection in rotating machinery.

**Figure 4:**
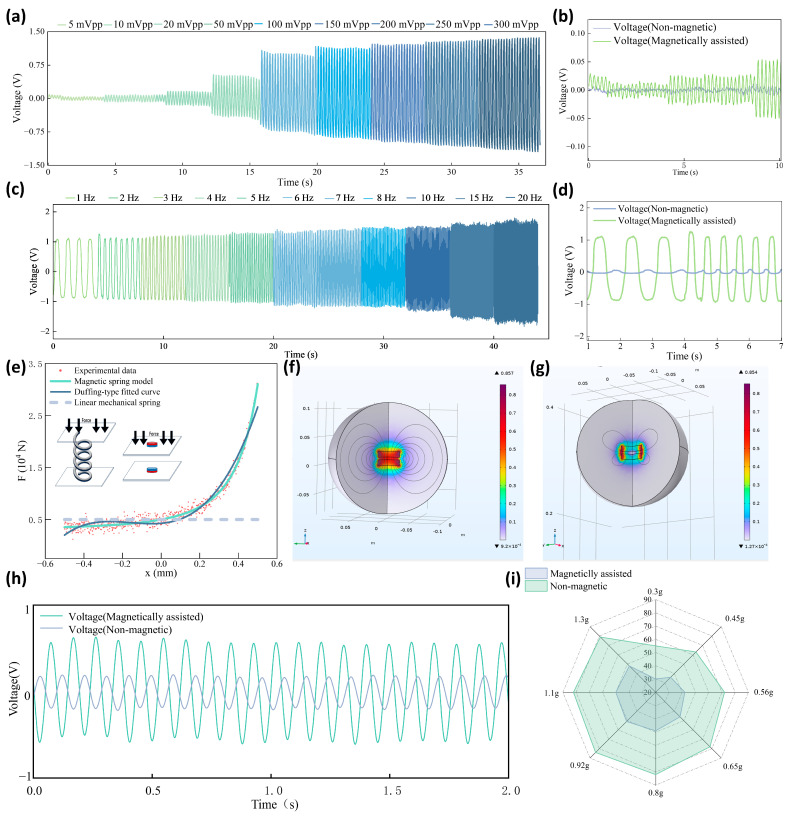
Vibration output performance of the MRCT and advantages of the magnetic-repulsion buffer structure. (**a**) Output voltage from the MRCT at different amplitudes under 5 Hz. (**b**) Comparison of the performance with and without the magnetic-repulsion buffer structure under weak excitation at the same frequency. (**c**) Output voltage of the MRCT at different frequencies with an amplitude of 150 mVpp. (**d**) Comparison of the magnetic-repulsion buffer structure and non-buffer structure under the same amplitude in the low-frequency range. (**e**) Force–displacement curve comparison between the traditional spring structure and the magnetic-repulsion buffer. (**f**) Magnetic field simulation of the magnetic-repulsion buffer structure. (**g**) Mechanical simulation of the magnetic-repulsion buffer structure. (**h**) Comparison of output voltage without a magnetic-repulsion buffer structure under the same imbalance weight in a real application. (**i**) Radar chart comparing the voltage output of the sensor with and without the magnetic-repulsion buffer structure at 8 Hz.

With the vibration frequency held at 5 Hz, the output voltage of the MRCT was measured as the excitation voltage increased from 5 mVpp to 300 mVpp (Figure 4a). At input voltages below 50 mVpp, the signal amplitude remained relatively low, although the output still showed stable periodic behavior. This is mainly because the contact–separation motion between the PDMS friction layer and the MSEA was weak under low excitation, resulting in limited charge transfer and therefore a small electrical output. Once the excitation voltage rose above 50 mVpp, the output amplitude increased noticeably. Between 50 mVpp and 200 mVpp, the magnetic-repulsion cushioning structure supplied additional restoring force, enabling stable and periodic deformation of the vibration system. At the same time, magnetic repulsion increased the effective separation distance between the friction layer and promoted a larger effective contact area of the microstructured strip electrode, which together enhanced charge transfer and improved signal output. When the excitation voltage exceeded 200 mVpp, contact and separation between the friction layer had become sufficiently complete, and the transferred charge no longer increased appreciably. As a result, the output amplitude gradually reached a saturation state.

### 3.2. Influence of the Magnetic-Repulsion Cushion on Sensor Performance

To better understand how the magnetic-repulsion structure affects the vibration response of the device, the output-voltage signals of the systems without magnets and with magnets were compared (Figures S3 and S4 in the Supporting Information). The results, shown in Figure 4b, indicate that without magnets, the output amplitude was relatively low, and the waveform showed evident attenuation and instability. After magnets were introduced, the signal amplitude increased markedly, and the waveform became more stable. Under weak excitation, the output amplitude gradually increased as vibration proceeded, while the signal maintained good periodicity. These observations suggest that the magnetic-repulsion structure effectively strengthens the contact–separation motion during vibration, thereby increasing both the friction layer contact area and the effective separation distance.

As shown in Figure 4c, when the excitation voltage was kept at 250 mVpp, the period of the output signal decreased gradually as the vibration frequency increased, and the output frequency remained consistent with the input excitation frequency. This result confirms the good frequency-tracking ability of the device. At low frequencies, the vibration system moved more slowly, and the contact–separation process between the friction layer was correspondingly slower, which led to a relatively low output amplitude. Figure 4d further shows that, with magnets, the output signal displayed clearer periodicity and stronger frequency-following behavior. This indicates that the magnetic-repulsion structure improves system stability and enhances the sensitivity and reliability of the MRCT in vibration monitoring.

To examine the influence of magnetic repulsion on the dynamic characteristics of the system, the restoring force–displacement relationship between the magnets was measured experimentally, and the resulting data were compared with the theoretical model, as shown in Figure 4e. The experimental results reveal a distinct nonlinear relationship between restoring force and displacement. At small displacements, the restoring force varies slowly, whereas it increases rapidly as the displacement becomes larger. This nonlinear feature arises from the repulsive force between the magnets, which depends inversely on the distance between them. As a result, the magnetic force changes more dramatically when the magnet spacing becomes small.

To characterize the nonlinear restoring force, the repulsive interaction between the magnets was approximated using Coulomb’s law for magnetic poles, according to which the force is inversely proportional to the square of the magnet spacing. The magnetic restoring force can therefore be expressed as an inverse-power function of distance. Taking into account the effect of vibration displacement x on the separation between the magnets, the magnetic-spring model is given by Equation (6):

(6) $$F_{mag}\left(x\right)=C\left[\frac{1}{{\left(d_{0}-x\right)}^{n}}-\frac{1}{{d_{0}}^{n}}\right]$$

where $d_{0}$ denotes the initial spacing, x is the displacement, C is a constant associated with the magnetic moment of the magnets, and n is the power exponent.

To further describe the experimental data, a polynomial expression analogous to the Duffing nonlinear vibration model was used, as shown in Equation (7):

(7) $$F\left(x\right)=k_{1}x+k_{3}x^{3}$$

where $k_{1}$ represents the linear stiffness and $k_{3}$ is the nonlinear stiffness coefficient. Polynomial fitting based on the Duffing model showed a high degree of agreement with the experimental results.

Compared with the conventional linear spring model, F(x)=kx, the results indicate that the magnetic-repulsion structure introduces a much stronger nonlinear restoring-force characteristic into the system. Through this nonlinear magnetic-spring effect, the effective stiffness can be tuned more effectively, while the dynamic response of the vibration system is also enhanced. This is beneficial for improving both the sensitivity and the energy-conversion efficiency of the MRCT in vibration monitoring. Figure 4f,g show the magnetic-field distribution and force analysis obtained from the COMSOL simulation, respectively. These results intuitively illustrate the operating mechanism of the magnetic-repulsion cushioning structure and provide theoretical support for sensor design.

To evaluate how the magnetically repulsive cushioning structure affects sensor performance, comparative experiments were conducted on two sensors, one equipped with magnets and the other without magnets. Vibration signals from the two TENGs were collected separately under the same imbalance-weight condition, and the corresponding results are presented in Figure 4h. Without magnetic repulsion, there is insufficient restoring force between the upper and lower friction layers. In this case, contact and separation rely mainly on the elastic deformation of the materials, which limits the effective contact area and separation distance and thus reduces interfacial charge-transfer efficiency. After magnets were introduced, the magnetic-repulsion structure was established, resulting in a clear increase in output amplitude and improved waveform stability.

To further compare the two designs, the voltage outputs of the sensor with the magnetic-repulsion cushioning structure and the sensor without magnets were measured under the same imbalance-weight condition, as shown in Figure 4i. The radar plot clearly indicates that the voltage output of the sensor with magnetic repulsion was consistently higher across the entire acceleration range. This confirms that the magnetic-repulsion structure effectively enhances sensor performance and improves both the sensitivity and reliability of the MRCT in vibration monitoring.

A broader comparison is provided in Table S3 in the Supporting Information, where the magnetic-repulsion MRCT is compared with the non-magnetic control device and commercial accelerometer sensors. The comparison further shows that the magnetic-repulsion design improves the sensitivity and weak-vibration response relative to the non-magnetic control device. Meanwhile, the MRCT retains the self-powered signal-generation feature of triboelectric sensing, which is useful for low-power and distributed vibration-monitoring scenarios.

### 3.3. Electrical Output Stability, Signal-to-Noise Ratio, and Frequency-Response Characteristics of the MRCT

**Figure 5:**
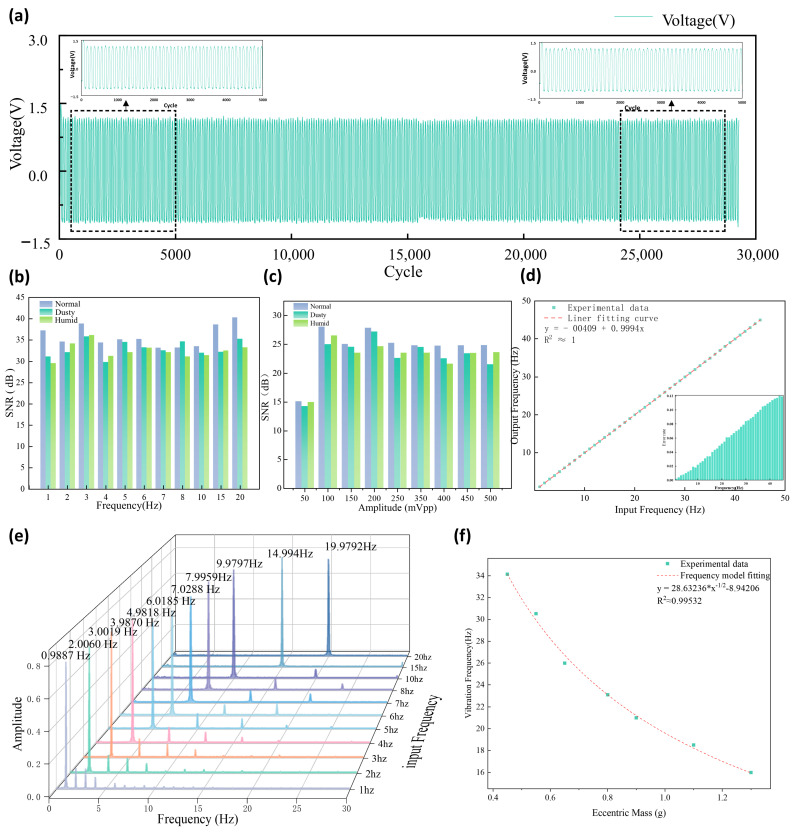
Sensing characteristics of the MRCT. (**a**) Stability test (30,000 cycles). (**b**) Signal-to-noise ratio (SNR) of the MRCT at different frequencies with an amplitude of 150 mVpp. (**c**) SNR of the MRCT at different amplitudes at a frequency of 1 Hz. (**d**) Frequency linearity of the MRCT in the range of 10–50 Hz. (**e**) Corresponding frequency spectrum of the MRCT in the 0–30 Hz frequency range. (**f**) Empirical relationship between the added imbalance weight and the dominant frequency component extracted from the MRCT voltage signal under the present fixed driving condition.

As shown in Figure 5a, the stability of the MRCT was evaluated at 25 Hz with an excitation amplitude of 200 mVpp. More than 30,000 operating cycles were recorded, and comparison of the waveforms from the first and last 5000 cycles showed no obvious change in the output signal. This indicates that the voltage waveform remained stable during prolonged operation, confirming the durability and reliability of the device. To further assess the self-sensing capability of the MRCT, the signal-to-noise ratio (SNR) was analyzed under different vibration frequencies and amplitudes. The results in Figure 5b,c show the SNR variation under normal, dusty, and humid conditions. In the frequency range of 1–20 Hz, the SNR remained at a relatively high level. Under normal conditions, the SNR reached about 35–40 dB. In dusty and humid environments, the SNR decreased slightly, but remained above 30 dB. This change is mainly attributed to altered interfacial contact in the presence of dust and to surface charge leakage under humid conditions, both of which can affect triboelectric charge accumulation. Even so, the SNR changed only slightly with frequency, indicating that the sensor maintains good stability and interference resistance in the low-frequency range.

Within the vibration-amplitude range of 50–500 mVpp, the SNR also showed a relatively stable trend under all three environmental conditions and remained between 20 and 30 dB. As the vibration amplitude increased, the SNR did not decrease significantly, suggesting that the sensor can maintain stable output under different excitation strengths. In particular, the SNR remained relatively high in the range of 100–200 mVpp. Taken together, these results show that the MRCT provides high SNR over a range of frequencies, amplitudes, and environmental conditions, indicating excellent signal quality and good environmental adaptability.

To further examine the frequency-tracking capability of the MRCT, the signal frequency measured by the MRCT was compared with the input frequency of the shaker and then subjected to linear fitting (Figure 5d). The experimental results show that, over the frequency range of 1–20 Hz, the measured signal frequency has excellent linearity with the shaker frequency. The fitted $R^{2}$ value is close to one, and the frequency-monitoring error is below 1.5%. As shown in Figure 5e, frequency-domain analysis of the x-channel signal over 1–20 Hz further indicates that the output frequency closely matches the excitation frequency.

To investigate the response pattern of the MRCT under different imbalance weights, the relationship between the motor vibration frequency f and the imbalance weight m was analyzed experimentally. The results show that the vibration frequency decreases clearly as the imbalance weight increases. Fitting of the data further reveals an inverse relationship between f and $\sqrt{m}$. In Figure 5f, the experimental data points agree closely with the fitted curve, indicating that this relationship can describe the dynamic behavior of the motor imbalance-vibration system with good accuracy.

To examine the frequency-related response under different imbalance-weight conditions, the dominant frequency component of the MRCT voltage signal was extracted and compared with the added imbalance weight, as shown in Figure 5f. In the initial analysis, the decrease in the dominant frequency was discussed using a mass–frequency relationship. However, the additional rotational-speed measurement showed that the motor speed also changed with the added imbalance weight. This result indicates that the observed frequency variation was strongly affected by rotational-speed variation and load change, rather than being caused only by a change in equivalent mass or structural natural frequency.

Therefore, the frequency change observed in this experiment is treated as an empirical feature of the motor–fixture–sensor system under the present driving condition. It is used here as an auxiliary feature for imbalance-state recognition, not as a direct physical basis for estimating imbalance mass. In rotating machinery, the primary vibration frequency is closely related to rotational speed, while the unbalanced force depends on the unbalanced mass, eccentricity, and the square of rotational speed. Direct inversion of imbalance mass from frequency alone would require synchronized speed measurement, eccentricity information, and a more complete dynamic model. The corresponding motor-speed measurements are provided in Table S4 in the Supporting Information.

### 3.4. MRCT-Based Vibration-State Recognition and Abnormal-Response Analysis

Rotating-machinery health-monitoring systems are important in industrial production, transportation, and new energy generation because they can improve operational safety, reduce structural fatigue, extend equipment service life, and lower the risk of accidents. To verify the practical effectiveness of the proposed sensor, a motor was selected as a demonstration case, and the MRCT was mounted on the motor assembly to monitor vibration under different imbalance-weight conditions. To enhance the noise immunity of the signal-conditioning circuit, a differential amplification circuit was introduced to shift and amplify the signal while keeping it within the range of 0–3.3 V. The corresponding circuit schematic is shown in Figure S5a in the Supporting Information. Vibration signals obtained under different imbalance-weight conditions were collected through this circuit and used to train the model for identifying motor vibration states.

**Figure 6:**
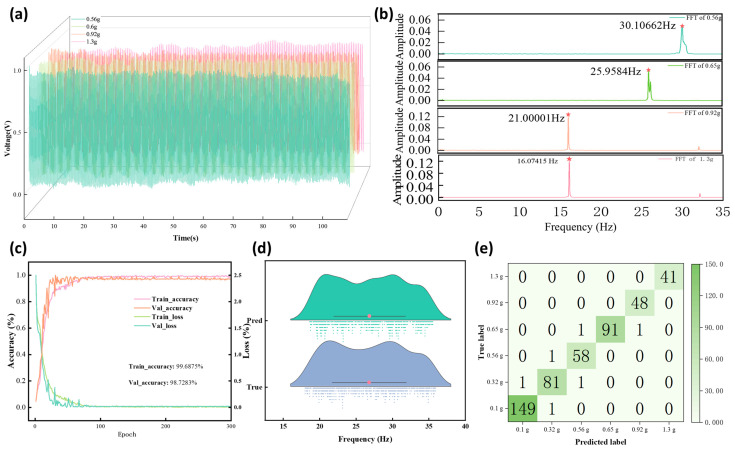
MRCT-based vibration signal characterization and deep-learning-assisted speed recognition of the vibration motor. (**a**) Output voltage signals collected by MRCT from the vibration motor under different imbalance weights. The variation in voltage amplitude reflects the change in vibration intensity induced by mass imbalance. (**b**) Frequency-domain spectra of the vibration signals under different imbalance weights, showing characteristic frequency components associated with different motor states. (**c**) Training accuracy and loss curves of the deep-learning model used for motor speed recognition. (**d**) Residual violin plot illustrating the distribution of prediction errors for speed recognition. (**e**) Confusion matrix evaluating the classification performance of the deep-learning-based speed recognition model.

According to the physical model established in Section 2.2, variations in external vibration affect the displacement response, contact-separation behavior, and charge-transfer process of the MRCT, which are reflected in the amplitude and periodicity of the output-voltage signal. Therefore, the time-domain waveform and frequency-domain response can be used to characterize different vibration states. As shown in Figure 6a, the voltage signal changes markedly with variation in imbalance weight, and the channel-signal frequency in Figure 6b also fluctuates clearly. Although the motor operates at a relatively high vibration frequency, the differences among the signals remain evident. Even so, direct discrimination from the time-domain waveforms alone is difficult. For this reason, the above CNN-GRU-based model was used for vibration-state recognition. The CNN-GRU model does not estimate the imbalance weight from the vibration frequency alone. Instead, it learns combined time-domain and frequency-domain features from the MRCT output, including signal amplitude, periodicity, waveform evolution, and spectral characteristics. Therefore, the reported imbalance-state recognition should be understood as data-driven classification of predefined operating states under the present experimental conditions, rather than analytical inversion of imbalance mass based only on frequency. The results show strong predictive performance. The results show strong predictive performance. The accuracy and loss curves in Figure 6c, the prediction-error density distribution in Figure 6d, and the confusion matrix in Figure 6e together indicate a training accuracy of 99.69% and a test accuracy of 98.73%. These results confirm that the model can effectively capture vibration-feature differences caused by changes in imbalance weight, thereby providing reliable support for motor-state identification.

In addition to the predefined imbalance-weight recognition task, a shaft-misalignment-related abnormal vibration condition was analyzed to examine the response capability of the MRCT to another representative abnormal state. Figure S5b shows the time-domain 3D waterfall plot of the MRCT voltage signals under normal and misalignment-related conditions. Compared with the normal state, the misalignment-related condition produces more pronounced waveform modulation and distinguishable amplitude variation, indicating that the MRCT can capture changes in vibration behavior caused by altered motor alignment.

Figure S6 in the Supporting Information presents the corresponding frequency-domain 3D waterfall plot. Under the misalignment-related condition, the spectral distribution becomes more complex than that under the normal state. Figure S7 in the Supporting Information further compares the local spectra around the 2× rotational frequency, where clear spectral variation can be observed. This result suggests that the MRCT voltage signal contains harmonic information associated with the misalignment-related abnormal vibration response, rather than only reflecting the overall vibration intensity.

These results support the capability of the MRCT to sense representative vibration-related abnormal states beyond rotor imbalance. However, the CNN-GRU model is mainly trained and evaluated for predefined imbalance-state recognition in this work, and a complete multi-fault diagnosis framework under variable-speed and cross-machine conditions requires further systematic investigation.

### 3.5. Real-Time AIoT Monitoring Demonstration

**Figure 7:**
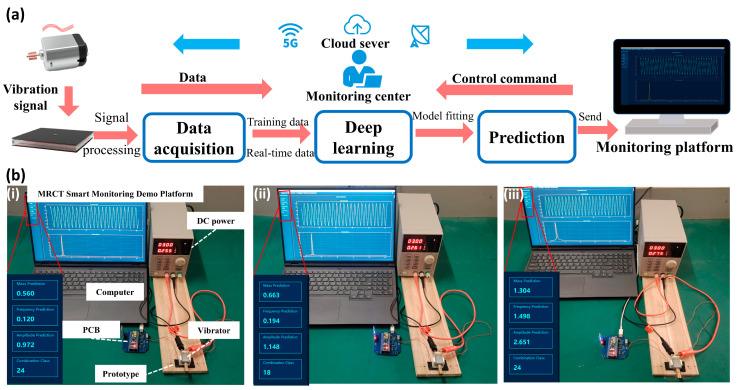
MRCT-based real-time vibration motor state monitoring system and online monitoring results. (**a**) Schematic diagram of the real-time monitoring platform based on MRCT, showing the workflow from vibration signal sensing and data acquisition to signal processing and motor state identification. (**b**) Real-time monitoring of the vibration motor under different imbalance weights, verifying the ability of the MRCT-based platform to identify motor operating states in real time: (**i**) monitoring interface and experimental setup under the 0.56 g imbalance condition; (**ii**) monitoring interface and experimental setup under the 0.65 g imbalance condition; (**iii**) monitoring interface and experimental setup under the 1.3 g imbalance condition.

To achieve remote visual monitoring in real-world applications, a rotating-machinery health-monitoring system based on the MRCT needs to be developed. The MRCT can act as a sensor node deployed in the physical environment, where multimodal data can be collected using an industrial AIoT system. Figure 7a illustrates the working principle of the MRCT-based rotating-machinery health-monitoring system. In the physical domain, a vibration motor is used to simulate the vibration of a vibrating screen in the corresponding virtual space. During the initial stage, the MRCT collects training data, which are used to train a deep-learning model. After model training and deployment, the system is capable of predicting the motor’s vibration state based on real-time signals and visualizing the results.

In the practical implementation, the trained deep-learning model is first loaded into the monitoring system. Real-time data are then input into the system for inference and analysis, with the results displayed in real time to present a visual representation of the machine’s health.

Figure 7b illustrates the application of the MRCT-based rotating-machinery health-monitoring system in a real-world experiment, with a video demonstration available in the Supporting Information. In the experiment, the MRCT was installed beneath a motor with an imbalanced weight to simulate a real-life scenario. The monitoring platform’s user interface displayed the operating state of the vibration motor in both time and frequency domains in real-time, allowing for efficient observation and analysis of the motor condition. By adjusting the imbalance weight, the motor output was controlled, simulating different imbalance states.

The experimental results indicate that the MRCT-based health-monitoring system can precisely identify the imbalanced weight of the vibration motor, thus enabling the evaluation of the motor’s rotational state under imbalance conditions and achieving the goal of remote monitoring and early warning. Additionally, if the MRCT is deployed as part of AIoT infrastructure in rotating systems such as automobiles, industrial machine tools, and wind turbines, it will enable real-time monitoring and fault diagnosis of rotating mechanical structures.

To further clarify the practical meaning of the real-time monitoring demonstration, the deployment conditions and system-level response metrics of the prototype were summarized in Table S5 in the Supporting Information. The present system was implemented on a PC-based laboratory platform, and wireless communication was realized using an HC-06 Bluetooth (commercially available, purchased from Taobao Marketplace, Hangzhou, China) serial communication module. Therefore, the demonstrated platform should be regarded as a laboratory-scale prototype for validating MRCT signal acquisition, CNN-GRU-based diagnosis, wireless transmission, and GUI-based state visualization, rather than a fully embedded or industrially deployed monitoring system.

As listed in Table S5 in the Supporting Information, the MRCT voltage signal was sampled at 100 Hz, and the CNN-GRU model used a 1000-point input window. Thus, one complete diagnosis window corresponded to 10.00 s of signal acquisition. The first alarm response was therefore dominated by the 10 s input window, rather than by the model inference time itself. After the initial buffer was filled, the sliding stride of two points theoretically allowed a prediction update interval of 20 ms, but the adjacent windows had a high overlap rate of 99.80%. The single-window inference time on the PC platform was conservatively estimated to be 8–15 ms, and the estimated Bluetooth latency was approximately 50–100 ms. Consequently, the estimated end-to-end alarm latency of the current prototype was approximately 10.10–10.20 s. These results indicate that the prototype is suitable for low-frequency rotor imbalance-state monitoring where second-level response is acceptable, while further optimization is required for industrial online monitoring scenarios requiring sub-second response.

Looking ahead, the MRCT may be integrated with low-power sensors, such as humidity and pressure sensors, to build a low-cost, intelligent, and sustainable monitoring ecosystem. In addition to its role in health monitoring, the MRCT can also function as a general vibration sensor. When combined with digital twins, 5G communication, and artificial intelligence, such sensors have the potential to be widely used in smart factories, intelligent power systems, smart transportation, and wearable devices. These technologies could also play a role in building self-sustaining digital-twin systems. The experimental results demonstrate that the MRCT excels in monitoring scope, accuracy, and real-time recognition performance.

## 4. Discussion

The MRCT developed in this study is intended as a self-powered vibration sensor for AI-assisted monitoring of rotating machinery. Rotor imbalance is a well-known vibration-related condition and, in many cases, can be identified using conventional acceleration measurement and frequency-domain analysis. Thus, this work does not aim to argue that imbalance is inherently difficult to diagnose. The main issue addressed here is different: how to obtain usable vibration signals with a simple, self-powered sensing structure that may reduce the need for external power supply and extensive wiring in distributed or hard-to-access monitoring locations. In this sense, the value of the MRCT lies in its sensing configuration, its response to weak low-frequency vibration, and its compatibility with low-power monitoring schemes. The comparisons in Tables S2 and S3 support this positioning. They do not imply that the MRCT should replace mature commercial accelerometers; rather, they show that the device is intended as a self-powered option for low-frequency distributed monitoring, where wiring and external power supply can increase system complexity.

The magnetic-repulsion cushion improves the mechanical response of the device. The non-contact restoring force helps the upper friction layer maintain a more regular contact–separation motion under weak excitation. Compared with the control device without magnetic repulsion, the MRCT produced a higher output amplitude and a more stable waveform, indicating that the magnetic cushion helps maintain effective triboelectric contact during vibration. The MSEA further increases the effective contact area and promotes charge generation. These results show that combining magnetic cushioning with microstructured electrodes is useful for improving low-frequency triboelectric vibration sensing.

The CNN-GRU model was used to examine whether the MRCT signals could support imbalance-state recognition. The classification results show that the generated voltage signals contain both time-domain and frequency-domain information related to different vibration states. However, the present study should be viewed as a proof-of-concept demonstration rather than a complete multi-fault diagnosis system. Other common abnormal conditions, such as mechanical looseness, shaft misalignment, bearing defects, and rubbing faults, may produce different waveform distortions, spectral components, and dynamic responses. These fault types were not included in the present experimental scope because the current platform was designed mainly for controlled imbalance-vibration tests. Introducing additional fault simulations without a standardized coupled-shaft or bearing-fault test rig could introduce uncontrolled boundary-condition changes and make the physical interpretation of the MRCT signal less reliable. Therefore, the current results should be interpreted as MRCT-based imbalance-state recognition under laboratory conditions. Future work will require a broader multi-fault dataset, controlled variable-speed operation, cross-machine validation, and long-term field testing to evaluate the generalization capability of both the MRCT-sensing device and the CNN-GRU model. The relationship between imbalance weight and dominant signal frequency also needs cautious interpretation. In rotating machinery, the dominant vibration frequency is closely linked to rotational speed, and it may also change with load and system dynamics. Therefore, the frequency-related features observed here are treated as empirical features under the present test conditions, not as a general rule for directly estimating imbalance mass.

The AIoT-oriented interface shows that the MRCT signal can be combined with data acquisition, model inference, wireless transmission, and visualization. At this stage, however, the system remains a laboratory-scale demonstration. As summarized in Table S5, the model was executed on a PC rather than on an embedded processor or cloud server, and wireless transmission was implemented using an HC-06 Bluetooth module. Although the raw single-channel MRCT data rate was low, repeated full-window transmission under a highly overlapped sliding-window strategy would not be suitable for the current Bluetooth prototype. In addition, Bluetooth packet loss was not systematically quantified under industrial electromagnetic interference, and the communication power consumption was estimated only for the Bluetooth module rather than for the complete monitoring system. The prototype operated continuously for 6 h without interruption in the laboratory environment, but multi-day or field-level industrial stability has not yet been demonstrated. Before practical industrial use, further work should focus on reducing the input window length, deploying the model on embedded hardware, quantifying packet loss and latency under industrial interference, evaluating the full-system power budget, and validating the system under different machines, fault types, rotational speeds, environmental disturbances, and long-term operating conditions.

Overall, the results support the feasibility of using a magnetically repulsive triboelectric sensor for self-powered low-frequency vibration sensing and AI-assisted imbalance-state recognition. The MRCT may be useful for distributed condition monitoring where wiring and power supply are limiting factors. Further work is still needed to clarify the physical interpretation of the signal features, broaden the fault dataset, and evaluate long-term reliability under industrial operating conditions.

## 5. Conclusions

In this study, a magnetically repulsive cushion triboelectric nanogenerator (MRCT) was developed for self-powered vibration sensing and rotor imbalance detection in rotating machinery. The integration of a magnetic-repulsion buffer structure and a microstructured strip electrode array enabled stable contact-separation motion, efficient charge transfer, and reliable electrical output under different vibration conditions. The experimental results confirmed that the MRCT can sensitively capture low-frequency vibration signals and distinguish rotor operating states under different imbalance masses. The device also showed good durability, maintaining consistent and repeatable output waveforms after 30,000 cycles.

A CNN-GRU-based identification method was further established to classify vibration amplitude, vibration frequency, and rotor imbalance mass. By combining MRCT output signals with time- and frequency-domain feature extraction, the proposed method achieved an identification accuracy of over 98% in both shaker-table tests and motor experiments with imposed imbalance masses. When integrated with an industrial AIoT platform, the system enabled real-time visualization, remote monitoring, and early warning of rotor operating states.

Overall, the proposed MRCT offers a feasible self-powered sensing strategy for intelligent structural health monitoring of rotating machinery. Future work will focus on improving the wear resistance of the triboelectric layer, enhancing environmental robustness, and expanding datasets from different machines and operating conditions to improve the generalization capability of the deep-learning model.

## Data Availability

The data supporting the findings of this study, including representative vibration-sensing signals, rotor imbalance datasets, and model-related results, are available in the Supplementary Materials. Additional data are available from the corresponding author upon reasonable request.

## Supplementary Materials

The following supporting information can be downloaded at: https://www.mdpi.com/article/10.3390/s26113587/s1, Supplementary File S1: Additional experimental data, raw sensing signals, model training results, and related supporting materials. References cited in the Supplementary Materials have been included in the main reference list [41,42,43,44,45,46,47,48,49,50,51].

## Abbreviations

The following abbreviations are used in this manuscript:

MRCT	Magnetically Repulsive Cushion Triboelectric Nanogenerator
TENG	Triboelectric Nanogenerator
MSEA	Microstructured Strip Electrode Array
AIoT	Artificial Intelligence of Things
CNN	Convolutional Neural Network
GRU	Gated Recurrent Unit
SNR	Signal-to-Noise Ratio
RNNs	Recurrent Neural Networks
