# Supplementary material for: Magnetically Repulsive Cushion Triboelectric Nanogenerator for Rotating Machinery Structural Health Monitoring
Source: Sensors (Basel). 2026 Jun 4;26(11):3587. doi: 10.3390/s26113587 (PMC13259471; doi:10.3390/s26113587)
Supplement: Supplementary file 1 [file sensors-26-03587-s001.zip › Supplementary File.pdf]

Supporting Information for Magnetically Repulsive Cushion Triboelectric Nanogenerator for Fault Diagnosis and Intelligent Health Monitoring of Rotating Machinery

Figure S1. Displacement response of the sensor under different excitation conditions (simulation).

The simulation illustrates the dynamic displacement variation of the magnetically repulsive structure under varying excitation amplitudes and frequencies.

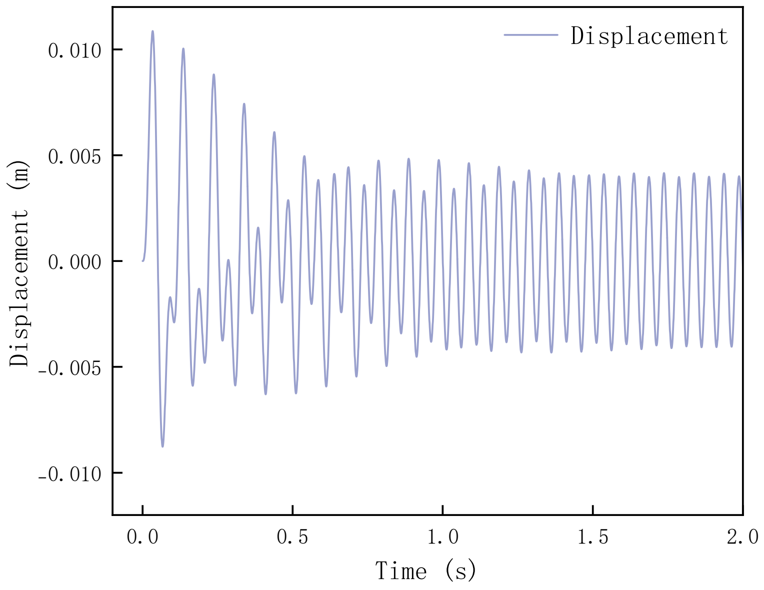

Figure S2. Corresponding frequency spectra of the displacement response.

The dominant frequency components reflect the vibration characteristics under different excitation conditions.

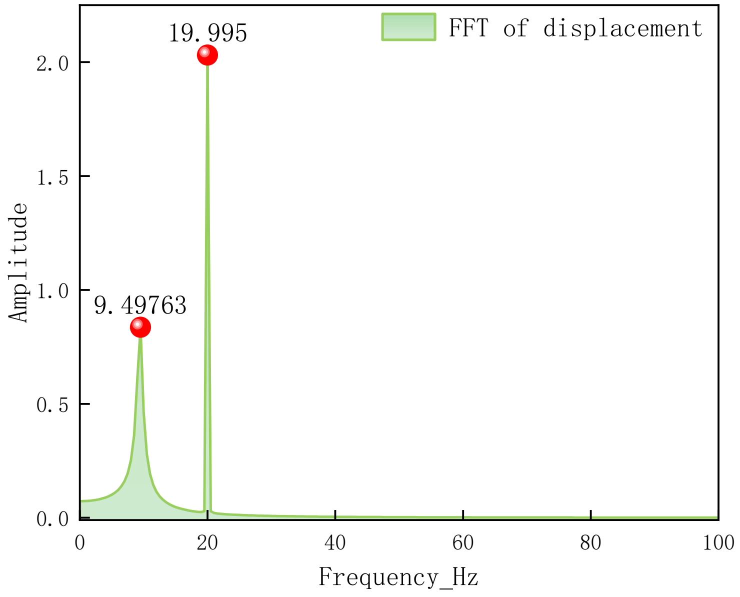

Figure S3. Output voltage of the TENG without magnets under different excitation frequencies.

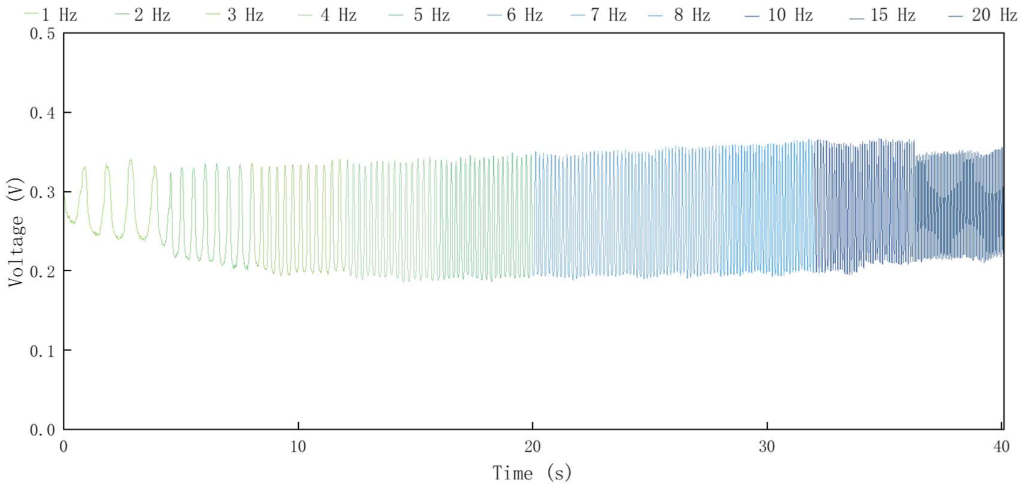

Figure S4. Output voltage of the TENG without magnets under different excitation amplitudes

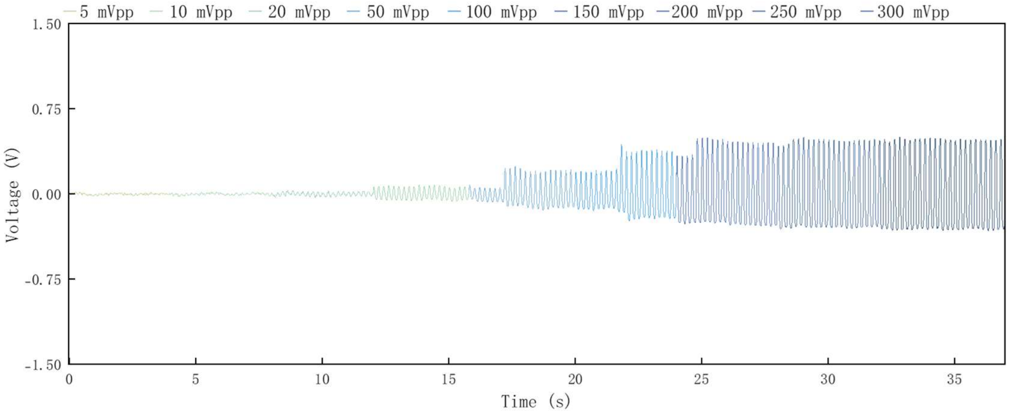

Figure S5a. Schematic of the multi-stage signal conditioning circuit for the TENG output.

The circuit amplifies and stabilizes the electrical signals generated by the TENG for subsequent data acquisition.

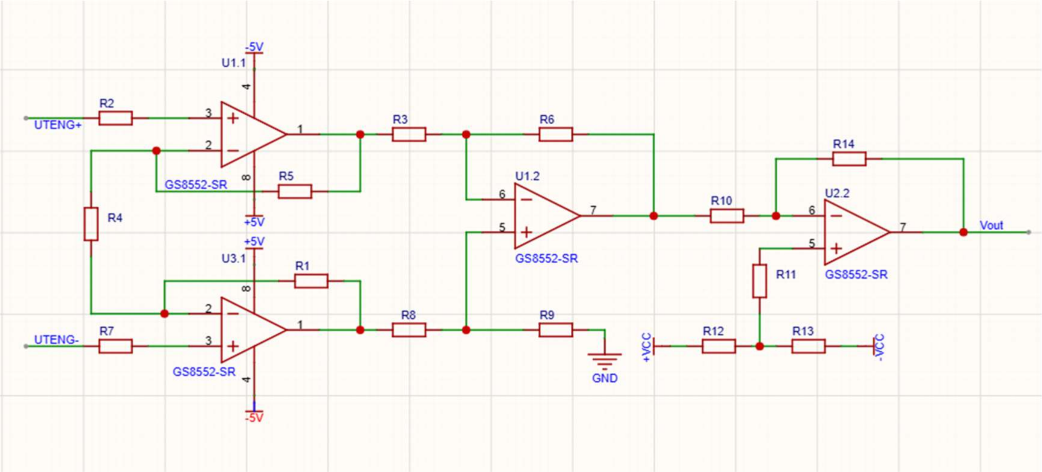

Figure S5b. Time-domain 3D waterfall plot of the MRCT voltage signal of the high-speed DC motor under normal and misalignment conditions.

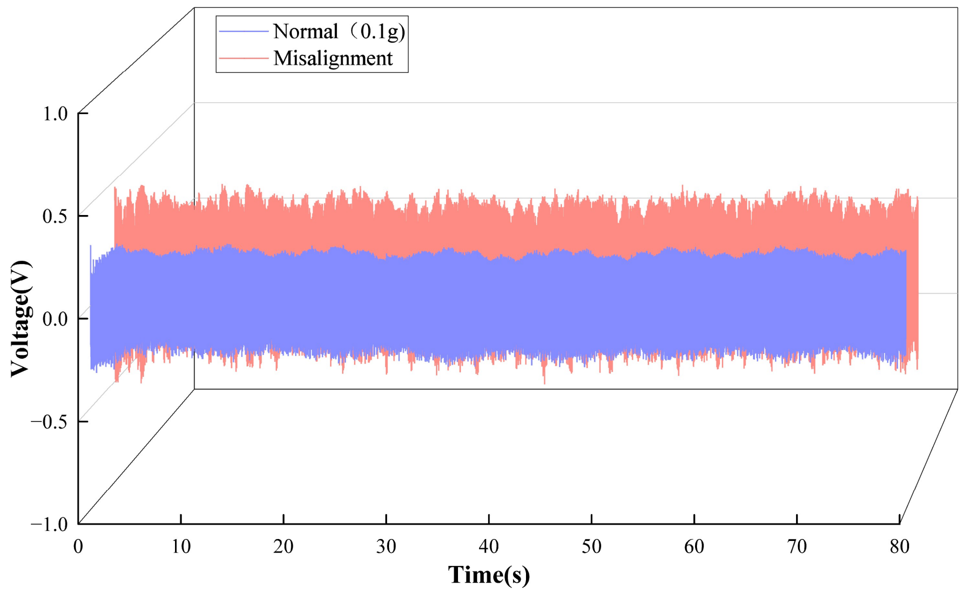

Figure S6. Frequency-domain 3D waterfall plot of the MRCT voltage signal under normal and misalignment conditions.

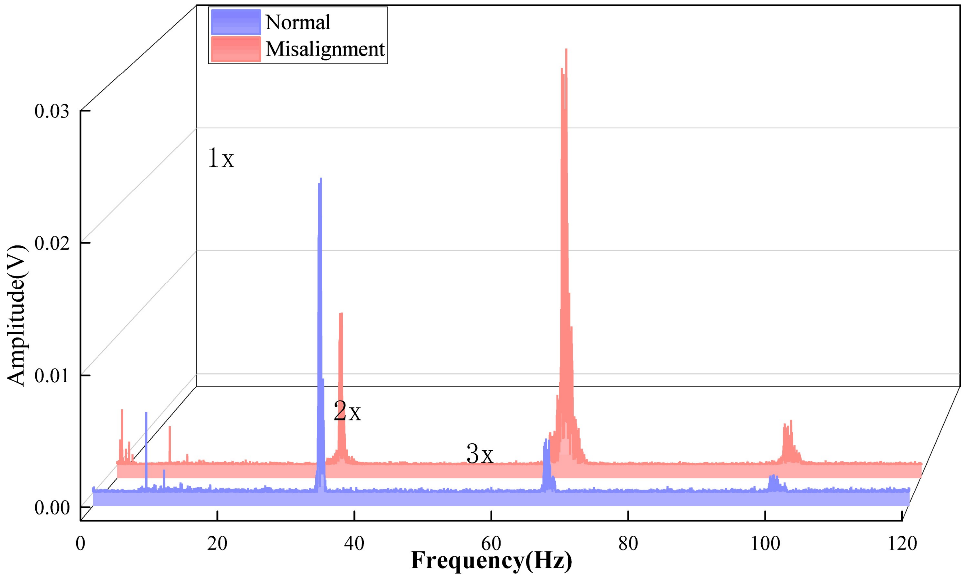

Figure S7. Local spectrum comparison around the 2× rotational frequency under normal and misalignment conditions.

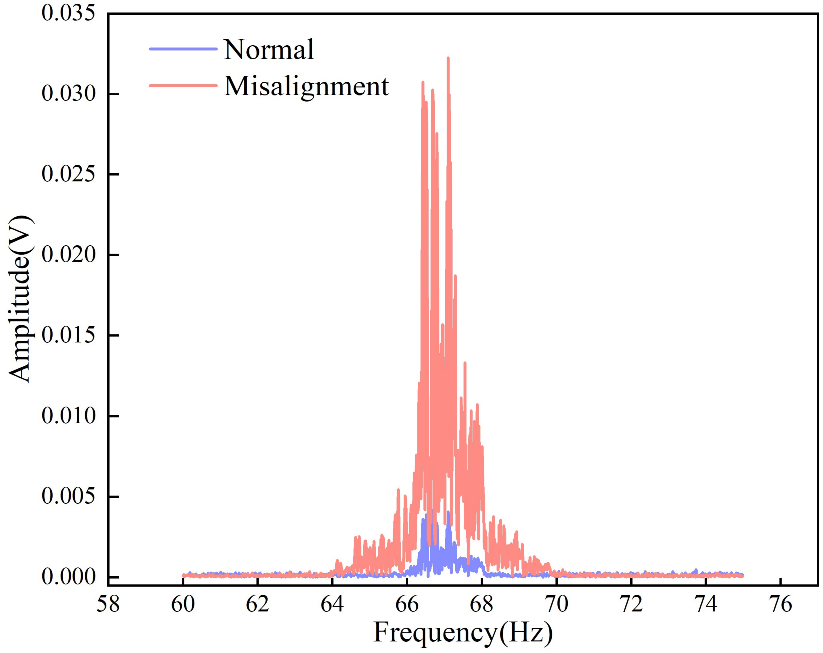

Table S1. Comparison with accelerometer sensors based on different working principles

Principle	Self-power	MEMS	Sensitivity	Sensitivity range	Flexibility	Reference
Capactive	No	Yes	187 fF/g		Yes	[1]
		No	0.197 pF/g	0.1-0.8 g	Yes	[2]
Piezoelectric	Yes	No	0.045V·s²/m	0.67-5.5 m/s²	Yes	[3]
		Yes	4.96 mV/g		No	[4]
		No	9.49 pC/g	0.5-20 g	No	[5]
Piezoresistive	No	No	1.67/(m/s²)	0.24-100 m/s²	Yes	[6]
		No		0.01-1.01 g	Yes	[7]
		No	0.0763 mV/g		No	[8]
Triboelectric	Yes	No	10.8 mV/g		No	[9]
		No		5-50 m/s²	Yes	[10]

		No	100 V/g	0.1-1.9 g	No	[11]
Triboelectric	Yes	No	4.39584mV/(m/s²)	0.5–50m/s²	Yes	This work

Table S2. Key design parameters of the MRCT and their effects on sensor characteristics.

Parameter	Value	Method	Effect
Magnet size	5 mm×0.5 mm	Caliper	Controls magnetic force
Magnet spacing	20 mm	Caliper	Adjusts restoring stiffness
Magnet arrangement	Repulsive face-to-face	Design	Enables non-contact cushioning
PDMS thickness	0.05 mm	Micrometer	Affects contact deformation
Effective contact area	400 mm²	Geometry calculation	Affects charge generation
MSEA strip width	2 mm	caliper	Affects contact area
MSEA strip spacing	2 mm	Optical image / caliper	Affects signal stability

Table S3. Comparison of the magnetic-repulsion MRCT, non-magnetic control device, and commercial accelerometer sensors.

**Index**	**This work**	**Without magnetic repulsion**	**ADXL335/ MPU6050**
Sensitivity	4.39584mV/(m/s²)	1.48753 mV/(m/s²)	10–30 mV/(m/s²)
Weak-vibration response (1–20 Hz)	SNR >30 dB	SNR ≈ 25–30 dB	Good, but external power required
Flexibility	Yes	Yes	No
Self-power	Yes	Yes, but lower output	No
Cost	Low	Low	High
Installation convenience	Yes	Simple attachment, but lower signal	No

			reliability	

Table S4. Rotational frequency under different imbalance-weight conditions.

Imbalance weight	Motor speed (rpm)	Rotational frequency (Hz)	Dominant frequency(Hz)	Main observation
0 g	20136	335.60	336.12	Stable high-speed baseline condition
0.32 g	19782	329.70	329.11	Slight rotational speed reduction induced by imbalance loading
0.56 g	19247	320.78	321.36	Dominant MRCT frequency follows rotational frequency variation
0.92 g	18431	307.18	306.47	Larger imbalance causes more evident load-related speed decrease

Table S5. Real-time performance and deployment conditions of the MRCT-based monitoring prototype

Parameter	Conservative value	Comment
Deployment platform	PC-based laboratory platform	Experimental validation only; not embedded or industrially deployed
Wireless communication	Bluetooth HC-06	Bluetooth 2.0 SPP serial communication module
Sampling frequency	100 Hz	Single-channel MRCT voltage signal
Window length	1000 points	Input length of the diagnosis model
Window duration	10.00 s	One complete window is required before the first prediction
Sliding stride	2 points	Adjacent windows are highly overlapped
Theoretical prediction update	20 ms	Sliding-window update interval after the initial 10 s buffer is

interval		filled
Window overlap rate	99.80%	Calculated from window length and stride
Raw data rate	0.39 kB/s, ≈3.13 kbps	Low raw sampling data rate
Full-window upload rate	195.31 kB/s, ≈1.56 Mbps	Calculated only; not suitable for the HC-06 prototype
Model architecture	CNN-GRU	Lightweight intelligent diagnosis model
Model parameters	129,478	Trainable parameters
FP32 model size	≈505.84 kB	Lightweight model size
Computational cost	≈28.35 M MACs/window	Estimated model complexity
Single-window inference time	8–15 ms	Conservative PC-based estimate
First alarm response time	≥10 s + processing and communication delay	Dominated by the 10 s input window
Estimated Bluetooth latency	50–100 ms	Conservative estimate for HC-06 short-range communication
Estimated end-to-end alarm latency	≈10.10–10.20 s	Dominated by the 10 s diagnosis window, plus Bluetooth and inference delay
Bluetooth packet loss	Not systematically quantified	No industrial interference test was conducted
Estimated packet loss	<1%	Short-range laboratory environment
HC-06 communication power	≈0.15–0.25 W	Estimated for the Bluetooth module only, not the whole system
Long-term stability	6 h continuous operation	No interruption observed in laboratory test
Industrial deployment	Not demonstrated	Current system is a laboratory prototype

References

[1].Mahmood, M.S., Z. Celik-Butler, and D.P. Butler, Design, fabrication and characterization of flexible MEMS accelerometer using multi-Level UV-LIGA. Sensors and Actuators A: Physical, 2017. 263: p. 530–541.

[2].Ye, J., et al., Tunable seesaw-like 3D capacitive sensor for force and acceleration sensing. npj Flexible Electronics, 2021. 5(1).

[3].Nour, E.S., et al., Low frequency accelerator sensor based on piezoelectric ZnO nanorods grown by low temperature scalable process. physica status solidi (a), 2016. 213(9): p. 2503–2508.

[4].Gong, X., et al., An aerosol deposition based MEMS piezoelectric accelerometer for low noise measurement. Microsystems & Nanoengineering, 2023. 9(1).

[5].Ding, Y., et al., Shear-structured piezoelectric accelerometers based on KNN lead-free ceramics for vibration monitoring. Journal of Materials Chemistry C, 2024. 12(46): p. 18639–18650.
[6].Chen, X., et al., Channel-Crack-Designed Suspended Sensing Membrane as a Fully Flexible Vibration Sensor with High Sensitivity and Dynamic Range. ACS Applied Materials & Interfaces, 2021. 13(29): p. 34637–34647.
[7].Korrapati, M., et al., Microstructured Scales in Porous Piezoresistive Vibration Sensor with Strain-Rate-Adaptive Gauge Factor. Macromolecular Materials and Engineering, 2022. 307(9).
[8].Yang, Y., et al., High-Temperature SiC Piezoresistive Accelerometer Fabricated by Femtosecond Laser. IEEE Sensors Journal, 2024. 24(11): p. 17461–17469.
[9].Qiao, Y., et al., Clapping triboelectric nanogenerators as self-powered, frequency-insensitive and gravity-independent vibration sensors. Nano Energy, 2023. 118.
[10].Zou, Y., et al., A Flexible, Adaptive, and Self-Powered Triboelectric Vibration Sensor with Conductive Sponge-Silicone for Machinery Condition Monitoring. Small, 2024. 20(32).
[11].Li, Y., et al., A non-contact triboelectric vibration sensor with a spiral floating electrode structure for low-frequency vibration monitoring. Nano Energy, 2025. 133.
